# Fabrication of atomically flat cleavage planes with ultrafast laser scribing

**DOI:** 10.1038/s41598-026-51163-0

**Published:** 2026-07-14

**Authors:** Francesco Scali, Wanyu Chen, Magnus H. Berntsen, Cong Li, Jacek Osiecki, Balasubramanian Thiagarajan, Dibya Phuyal, Maciej Dendzik, Oscar Tjernberg

**Affiliations:** 1 Dipartimento di Fisica, Politecnico di Milano, Piazza Leonardo da Vinci 32, 20133 Milano, Italy; 2 Department of Applied Physics, KTH Royal Institute of Technology, 11419 Stockholm, Sweden; 3 MAX IV Laboratory, Lund University, 22100 Lund, Sweden

**Keywords:** Materials science, Optics and photonics, Physics

## Abstract

The preparation of extensive, atomically flat surfaces remains a central challenge in modern quantum materials research, as many crystals lack natural cleavage planes suitable for advanced surface-sensitive investigations. Here, we demonstrate that laser scribing guided by an ultrafast laser can be applied to facilitate easy cleavage along a desired crystallographic plane under ultra-high vacuum. The method is validated on two brittle materials, $\textrm{SrTiO}_3$ and Si. The technique allows precise spatial localization of the cleaving site and produces extensive, uniformly oriented, and atomically flat surfaces, as verified by scanning electron microscopy (SEM) and atomic force microscopy (AFM). When applied to $\textrm{SrTiO}_3$, the technique enables angle-resolved photoemission spectroscopy (ARPES) measurements of surface electronic states characteristic of the two-dimensional electron liquid (2DEL) hosted at its bare (100) surface. Moreover, ultrafast laser scribing is significantly faster than focused ion beam (FIB) techniques for preparing cleavable planes, offering a more accessible and efficient approach. Owing to its broad applicability, this method establishes a powerful and general framework to prepare high-quality surfaces for advanced photoemission and microscopic investigations of quantum phenomena.

## Introduction

Extensive and atomically flat surfaces are crucial for investigations of quantum phenomena using surface-sensitive techniques, such as ARPES^1–3^ and scanning tunneling microscopy (STM)^4,5^. Materials with natural cleavage planes are particularly suitable for such experiments, as their weak interlayer bonding allows the preparation of high-quality surfaces through cleaving^2,3,6–8^. Unfortunately, only a limited number of crystals exhibits natural cleavage planes, typically restricted to specific crystallographic orientations^1–3,9^. Such cleavage emerges only when bonding is strongly anisotropic, creating low-energy planes that preferentially fracture; examples include layered van der Waals materials and ionic crystals. By contrast, many technologically relevant oxides and strongly covalent systems possess more isotropic bonding and therefore fracture irregularly rather than along predictable planes. Consequently, truly clean and easily accessible cleavage is relatively rare and confined to a small subset of crystal structures. This limitation has motivated the development of alternative methods to induce non-natural cleavage while maintaining high-quality surfaces.

In recent years, several mechanical crystal cleavers designed for *in situ* (i.e., under ultra-high vacuum) operation have been proposed^10–12^. Although these methods are effective in certain cases, the devices typically rely on blades and bulky anvils to cut and support the crystals, making them difficult to implement or transport between experimental setups. More recently, advanced approaches based on xenon-plasma FIB machining^13^ and strain-assisted cleaving^14^ have been successfully employed to achieve controlled cleavage of cubic perovskite oxides, such as $\textrm{SrTiO}_3$. In particular, the use of strain-assisted cleaving has enabled detailed investigations of surface electronic states associated with the 2DEL formed at the bare $\textrm{SrTiO}_3$ surface, offering new opportunities to elucidate the microscopic origin of these states^15^. However, strain-assisted cleaving devices, while particularly effective for cubic perovskites, are not readily transferable to other hard-to-cleave crystals. Similarly, the widespread adoption of xenon-plasma FIBs remains limited by their accessibility and operational speed, as the fabrication of a ready-for-cleave sample requires a few machining hours^13^. Although more common, conventional gallium FIBs are inadequate alternatives because they lack the high-current milling capability required to process samples on the scale of hundreds of micrometers.

In the present work, we introduce a novel approach to direct non-natural cleavage based on ultrafast laser scribing, in which a femtosecond-pulsed laser locally generates a micrometer-scale groove through the delivery of intense ultrashort laser pulses, thereby inducing localized ablation that enables subsequent controlled cleavage. Unlike previous techniques, we provide here a broadly applicable method for the fabrication of high-quality surfaces that remain spectroscopically unexplored. The approach relies on a tunable laser setup that provides control over key experimental parameters, including wavelength, pulse energy, operating position, scan velocity, and number of passes. While the wavelength determines the electron kinetic energy after photon absorption, and thus ionization processes at the base of ablation^16^, the pulse energy and operating position affect the fluence on the target and whether the ablation threshold is exceeded^17^. The scan velocity and number of passes control the exposure time under the laser beam, which affects the collateral damage and the groove depth. Therefore, these parameters can be optimized to induce ablation and achieve a groove deep enough to guide cleavage. Moreover, the underlying ablation mechanisms are shared among a broad spectrum of elements and compounds, including non-conductive materials that are difficult to process with FIB. Furthermore, ultrafast laser scribing is a non-contact method, avoiding sample contamination from machining tools, and is highly efficient, allowing tens of samples to be prepared for cleavage within an hour. Femtosecond-pulsed lasers are increasingly available in quantum materials laboratories, providing an accessible platform for implementing this approach. To demonstrate its effectiveness and flexibility, the method is validated on both $\textrm{SrTiO}_3$ and Si single crystals.

## Results and discussion

### Ultrafast laser scribing

In our approach, ultrafast laser scribing creates a groove, concentrating intense laser pulses in a micrometer-sized region of the workpiece over a very short duration. This rapid energy exposure leads to impact ionization and strong electric-field ionization of hot electrons^17^. Then, electrons transfer energy to the lattice due to electron-phonon interaction, raising the temperature above the melting point and causing ablation by thermal vaporization. These mechanisms are common across different elements and compounds, although their thresholds and efficiencies may vary^16,18^, leading to a universal material removal process^16–20^. This locally breaks the interlayer bonding, thereby facilitating controlled cleavage even along planes traditionally considered difficult to cleave. The lattice melts in a short time scale (*∼* ps) after the ultrafast laser pulse, thereby minimizing the heat load to the surrounding material and thermal damage in the target^17^. This is particularly crucial in brittle materials to avoid crack formation^18^.

**Fig. 1:**
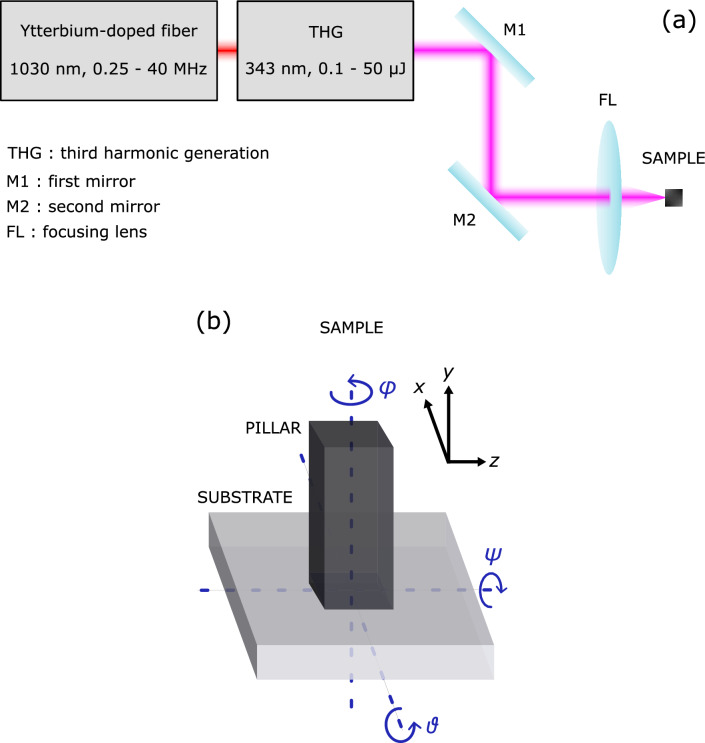
(**a**) Setup for ultrafast laser scribing based on a femtosecond-pulsed ytterbium-doped fiber laser equipped with a THG module. (**b**) Schematic of the sample, consisting of a pillar glued to a substrate and mounted on a six-degree-of-freedom holder. Grooves are produced by automatically scanning along the *x* axis the entire lateral surface of the pillar beneath the laser beam.

The laser scribing setup is shown in Fig. 1a. An ultrafast ytterbium-doped fiber laser (Tangerine, Amplitude Laser) generates *∼* 300 fs pulses, at a central wavelength of 1030 nm and a pulse repetition frequency tunable from 250 kHz to 40 MHz. The system is equipped with a third harmonic generation (THG) module, enabling operation with a pulse energy tunable up to 50 ${\mu \textrm{J}}$ at a central wavelength of 343 nm to maximize $\textrm{SrTiO}_3$ light absorption. The beam is focused onto the sample using two ultraviolet-centered broadband dielectric mirrors, each set at a $45^\circ$ angle of incidence, and an objective lens with a 30 mm focal length. The sample presented in Fig. 1b, comprising a pillar glued to a substrate, is mounted on a six-degree-of-freedom holder, allowing automatic movement along the *x* and *y* axes, manual alignment along the *z* axis, and manual adjustment of the *ψ*, *φ*, and *θ* angles. The pillars are the target of investigation and they are prepared as described in Sec. 3. The operating position along the *z* axis corresponds to the lens focal point, identified by increasing the pulse energy above 20 ${\mu \textrm{J}}$ to induce plasma formation in air at the location of minimum beam spot size. Along the *y* axis, the pillar is positioned at the desired scribing site, while along the *x* axis it is scanned over its entire lateral dimension beneath the laser beam to create the groove.

### SEM and AFM characterization of $\textbf{SrTiO}_3$

$\textrm{SrTiO}_3$ is a perovskite oxide with a cubic unit cell at room temperature in the absence of applied strain. It serves as a key platform in the emerging field of oxide electronics, offering pathways to overcome the scaling limitations of CMOS technology in information processing^21,22^. Moreover, its cubic symmetry and lattice constant make $\textrm{SrTiO}_3$ an ideal substrate for epitaxial growth using techniques such as pulsed laser deposition (PLD) and molecular beam epitaxy (MBE), owing to its excellent lattice matching with a wide range of oxide materials^23,24^. Heterostructures based on $\textrm{SrTiO}_3$ have been shown to host emergent phenomena such as high-temperature superconductivity^25,26^ and pronounced negative magnetoresistance^27^. These effects originate from the collective behavior of electrons driven by strong electron-electron interactions at the interface, revealing that the interfacial two-dimensional system forms a correlated electron liquid rather than a non-interacting electron gas. $\textrm{SrTiO}_3$ is also known for its extreme brittleness and typically exhibits a conchoidal fracture under uncontrolled conditions^9^.

**Fig. 2:**
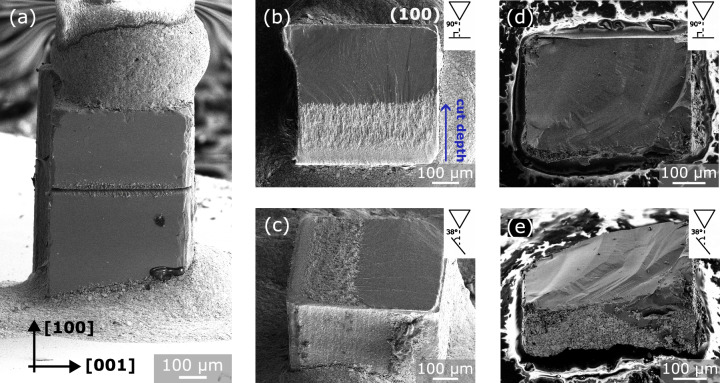
(**a**) SEM image of the groove scribed along the [001] crystallographic axis of a $\textrm{SrTiO}_3$ pillar oriented along the [100] direction. It exhibits a uniform lateral width of approximately 20 ${\mu \textrm{m}}$, maintained consistently along the entire scribed path. SEM images of the corresponding cleaved surface parallel to the (100) plane are acquired with the electron beam (**b**) incident normal to the sample and (**c**) at a polar angle of $\theta = 38^\circ$. In (**b**), the upper smooth surface exhibits extensive, well-cleaved regions that share a uniform crystallographic orientation, while the lower rough surface damaged by the laser delineates the cut depth. SEM images of a prototypical surface obtained by cleaving without any particular technique are shown in (**d**) and (**e**), where the resulting morphology shows a markedly inferior quality.

Figures 2a-c present scanning electron microscopy (SEM) images of a groove scribed on a $\textrm{SrTiO}_3$ pillar oriented along its [100] crystallographic axis and the corresponding cleaved surface, fabricated as reported in Sec. 3. The groove scribed along the [001] axis (Fig. 2a) demonstrates the precision achievable with ultrafast laser scribing. It exhibits a uniform lateral width of approximately 20 ${\mu \textrm{m}}$ maintained consistently along the entire scribed path. Its uniformity and the absence of cracks reflect the control afforded by femtosecond-pulsed laser, where fast energy deposition confines material ablation to micrometer-scale dimensions without inducing collateral damage to the surrounding regions. The cleaved surface (Figs. 2b and c), with dimensions of $500 \times 500\,{\mu \textrm{m}}^2$, confirms that the cleavage occurs precisely where the groove was induced, providing physical control over the cleaving location. The amorphous area on the bottom results from laser ablation and defines a cut depth of approximately $200\,{\mu \textrm{m}}$. The surface on the top exhibits extensive, well-cleaved regions separated by rough micrometer-scale steps. These regions extend laterally over more than $100\,{\mu \textrm{m}}$ and share a single crystallographic orientation, confirming that the cleavage plane is the (100) across the entire surface. Their lateral dimension exceeds the typical photon beam spot size used in ARPES measurements^1–3^, ensuring that the probed area can be contained within one macroscopic domain and thereby minimizing artifacts related to surface inhomogeneity. The achieved cleavage sizes are the largest reported for $\textrm{SrTiO}_3$, comparable only to those obtained using strain-assisted cleaving^14^.

Figures 2d and e present SEM images of the surface obtained by cleaving without any specific technique. As can be observed, the resulting morphology exhibits a markedly inferior quality when compared with the laser-scribed case. In particular, the surface is noticeably tilted and displays several regions that are misoriented with respect to each other, thereby indicating the absence of a well-defined and continuous crystal orientation. Consequently, the fracture plane cannot be associated with a single crystallographic plane, but rather corresponds to a heterogeneous combination of differently oriented domains.

**Fig. 3:**
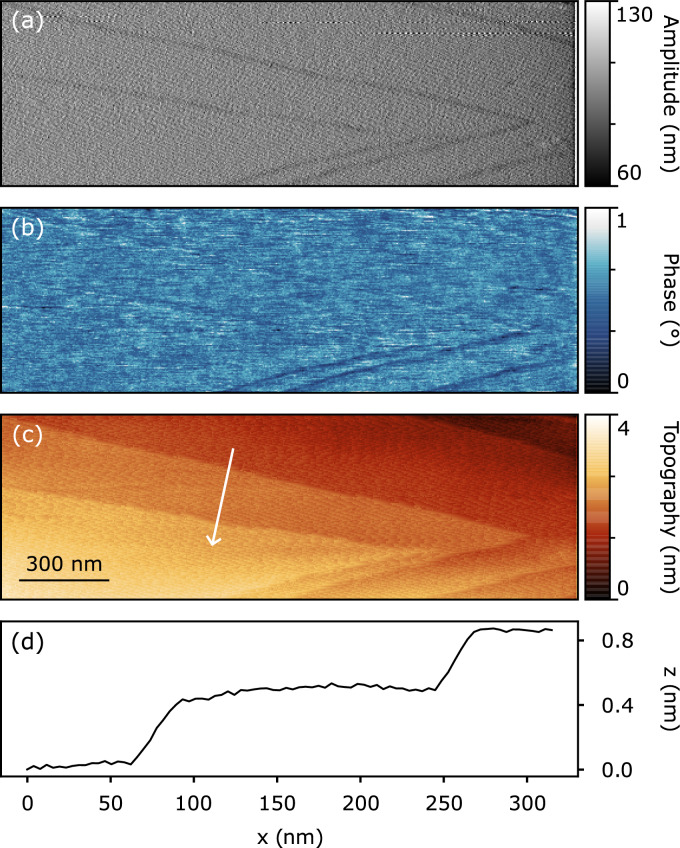
Tapping-mode AFM images of the (**a**) amplitude and (**b**) phase of the cantilever oscillation, and (**c**) the surface topography scanned under ambient conditions over the $\textrm{SrTiO}_3$ (100) cleaved surface. (**d**) Line profile taken along the white arrow in (**c**), highlighting the step height between atomic terraces, which matches the $\textrm{SrTiO}_3$ lattice constant.

Figures 3a -c show tapping-mode atomic force microscopy (AFM) images acquired within one of the homogeneous cleaved regions on the $\textrm{SrTiO}_3$ (100) surface identified in Figs. 2b and c. The topography image (Fig. 3c) reveals atomically flat terraces with lateral dimensions exceeding 100 nm, separated by monoatomic steps. The amplitude and phase maps of the cantilever oscillation (Figs. 3a and b), which are sensitive to local variations in tip-sample interactions, exhibit notable spatial uniformity, confirming the flatness and cleanliness of the region. A representative line profile (Fig. 3d), crossing the terraces and extracted along the white arrow in Fig. 3c, shows step heights of approximately 0.4 nm, in excellent agreement with the lattice constant of $\textrm{SrTiO}_3$. Remarkably, the surface exhibits an exceptionally small miscut angle, below $0.2^\circ$, matching the tolerance range of commercially available substrates used in thin-film growth. The atomically flat terraces observed by AFM are uniformly oriented and do not correspond to different crystallographic domains, since the cleaved regions originate from a single crystal. ARPES can therefore be performed in the cleaved regions despite containing several terraces, without the need for nano-ARPES.

### ARPES measurements on $\mathbf {SrTiO_3}$

**Fig. 4:**
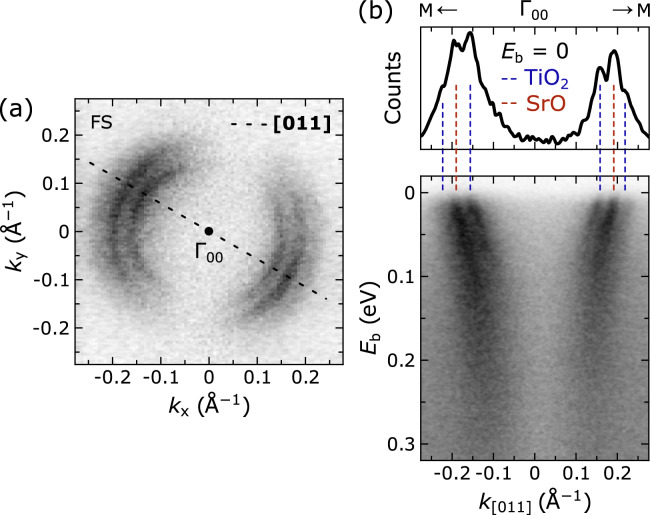
(**a**) Fermi surface (FS) of $\textrm{SrTiO}_3$, showing multiple quantum well states associated with the 2DEL hosted at its bare (100) surface. (**b**) Valence band dispersion along the *Γ*M high-symmetry direction and the momentum distribution curve cut at the Fermi level, displaying peaks associated with surface electronic states from both $\textrm{TiO}_2$ and $\textrm{SrO}$-terminated regions.

Figures 4a and b present ARPES measurements acquired as described in Sec. 3 on a $\textrm{SrTiO}_3$ (100) surface prepared identically to the one previously described. The Fermi surface of $\textrm{SrTiO}_3$ (Fig. 4a) consists of multiple quantum well states associated with the 2DEL hosted at its bare (100) surface^15,28–32^. The double-ring pattern is attributed to contributions from both $\textrm{TiO}_2$- and $\textrm{SrO}$-terminated surfaces. Specifically, the inner band originates from the $\textrm{TiO}_2$ termination, while the outer band from the $\textrm{SrO}$ termination. Indeed, in the momentum distribution curve (MDC) (Fig. 4b) cut at the Fermi level along the *Γ*M high-symmetry direction, the momentum-space separation between the peaks of the double ring is 0.035 Å$^{-1}$. This value is in excellent agreement with the momentum-space separation between $\textrm{TiO}_2$ and $\textrm{SrO}$ contributions calculated from the MDCs reported in Ref.^[15]^, of approximately 0.036 Å$^{-1}$. In addition, two weaker third peaks appear at larger absolute Fermi momenta. The latter are attributed to a second contribution from the $\textrm{TiO}_2$ surface termination. Actually, the momentum separation of these weaker third peaks from the inner peaks of the double ring perfectly matches the value of 0.067 Å$^{-1}$, calculated from Ref.^[15]^ and Ref.^[32]^, between bands originating from $\textrm{TiO}_2$-terminated surfaces.

Concerning the origin of these states, we observed that the 2DEL-related bands emerge within a few seconds of synchrotron illumination, consistent with previous reports^15,28–31^. For the SrO termination, the formation of the 2DEL is driven by the creation of positively charged oxygen vacancies, which induce downward band bending below the Fermi level and trigger an insulator-to-metal transition. In contrast, the TiO$_2$ termination exhibits metallic behavior immediately after cleaving, without the need for defect formation^15^. The additional spectral contribution associated with the TiO$_2$ termination originates from a Zeeman-type splitting of the 2DEL bands, arising from inversion symmetry breaking at the surface due to Ti-atom displacements combined with the emergence of unbalanced spin textures^15^.

**Fig. 5:**
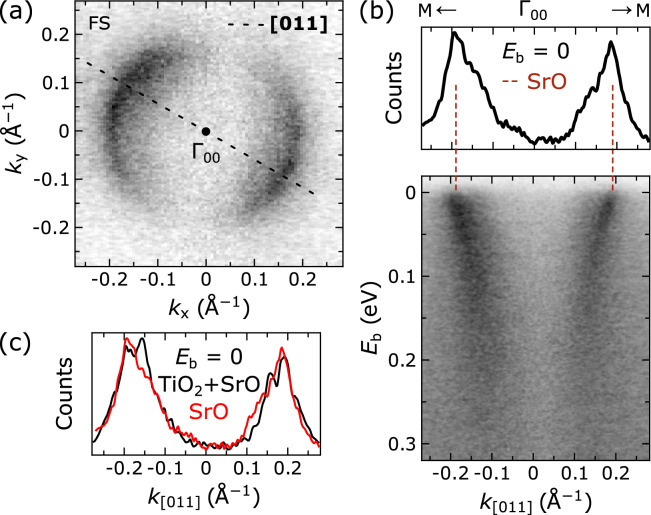
(**a**) Fermi surface (FS) of $\textrm{SrTiO}_3$ (100) observed while illuminating a $\textrm{SrO}$-terminated area on the sample. (**b**) Valence band dispersion along the *Γ*M high-symmetry direction and the momentum distribution curve cut at the Fermi level, displaying two single peaks for positive and negative Fermi momenta, respectively. (**c**) Comparison between the MDCs measured while illuminating the first position, exhibiting both the $\textrm{TiO}_2$ and $\textrm{SrO}$ terminations, and the second position, exhibiting only the $\textrm{SrO}$ termination.

The investigation of a different position on the very same $\textrm{SrTiO}_3$ (100) surface is presented in Figs. 5a-c. The observed single-ring shaped Fermi surface (Fig. 5a) and the corresponding peaks in the MDC (Fig. 5b), also cut at the Fermi level along the *Γ*M high-symmetry direction, are characteristics of the $\textrm{SrO}$ termination. The Fermi momenta of the peaks closely match the values reported in Ref.^[28]^ and furnish a way to separate the contributions coming from different terminated surfaces. The comparison between the MDCs obtained while illuminating the two positions (Fig. 5c) highlights the characteristic spectral features associated with each surface termination and supports the presented interpretation. Detecting such distinct signatures from multiple terminations within a single sample confirms that the cleaved surface is atomically well-defined.

### SEM and AFM characterization of $\textbf{Si}$

To demonstrate the general applicability of the technique, we applied the same workflow to silicon as a second test case. The latter is the foundation of conventional electronics and has also attracted interest for novel spintronics applications, motivated by recent spin transport studies^33–36^. It is a brittle crystal that crystallizes in a covalent structure with a diamond cubic lattice. Despite the presence of low energy planes, micro-cleaving workstations typically equipped with diamond scribe tips and optical microscopes are still required to align and pre-cut the material precisely along these easy axes in order to have high-quality cleavage under ambient conditions^37,38^. Therefore, it serves as a suitable benchmark for assessing the versatility of the technique.

**Fig. 6:**
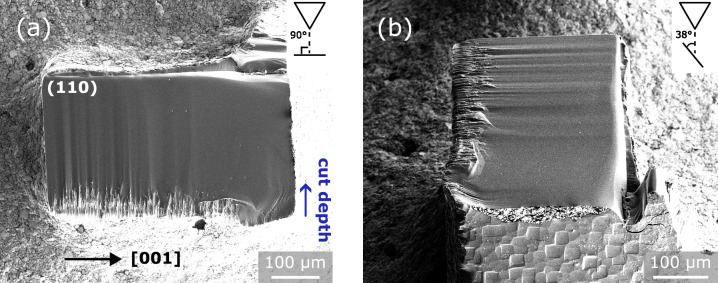
SEM images of the Si cleaved surface parallel to the (110) plane, acquired with the electron beam (**a**) incident normal to the sample and (**b**) at a polar angle of $\theta = 38^\circ$. The sample is scribed along the [001] crystallographic axis. The blue arrow in (**a**) indicates the cut depth.

Figures 6a and b present SEM images of a prototypical cleaved surface obtained on a Si pillar oriented along the [110] crystallographic axis prepared as explained in Sec. 3. In this case as well, the cleavage occurs precisely along the laser-induced groove. The cleaved surface, measuring $300 \times 500\,{\mu \textrm{m}}^2$, exhibits extensive, well-cleaved regions separated by unidirectional micrometric corrugations. These regions preserve a single crystallographic orientation along the [110] direction and extend laterally beyond $100\,{\mu \textrm{m}}$, comparable to the ones obtained for $\textrm{SrTiO}_3$. Notably, the ablated area is reduced compared with the $\textrm{SrTiO}_3$ case, with a shallow cut depth of approximately $50\,{\mu \textrm{m}}$. This could be attributed to the lower interplanar energy of the (110) plane in Si compared to the (100) plane in $\textrm{SrTiO}_3$.

**Fig. 7:**
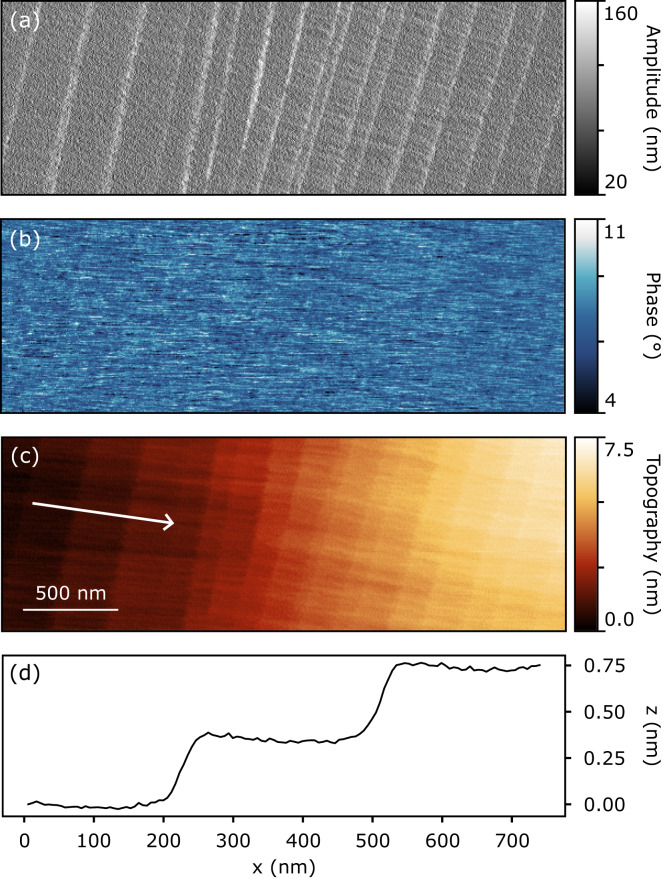
Tapping-mode AFM images of the (**a**) amplitude and (**b**) phase of the cantilever oscillation, and (**c**) the surface topography scanned under ambient conditions over the Si (110) cleaved surface. (**d**) Line profile taken along the white arrow in (c), showing the step height between adjacent atomic terraces which is consistent with the interplanar spacing of successive Si (110) planes.

Figures 7a-c show tapping-mode AFM images acquired over a well-cleaved region of the Si (110) surface shown in Figs. 6a and b. The topography (Fig. 7c) reveals atomically flat terraces with lateral dimensions exceeding 200 nm. Within these terraces, the amplitude and phase maps of the cantilever oscillation (Figs. 7a and b) appear homogeneous, confirming the absence of structural defects. A representative line profile (Fig. 7d), extracted along the white arrow in Fig. 7c, shows step heights of approximately 0.38 nm, in excellent agreement with the interplanar spacing of successive Si (110) planes^39^. As for $\textrm{SrTiO}_3$, the surface displays a miscut angle below $0.2^\circ$, within tolerances of metrology-grade substrates.

These results collectively establish ultrafast laser scribing as a technique that achieves atomically precise surface preparation along non-natural cleavage planes, and enables direct spectroscopic access to state-of-the-art 2DEL-related phenomena. The resulting morphology is exceptionally suited for advanced spectroscopic and microscopic studies, as demonstrated by SEM, AFM and ARPES measurements. Among the materials whose investigation could be facilitated by this method, we mention strongly correlated electron systems, such as cuprate and iron pnictide superconductors^1,40^, materials with strong spin-orbit coupling^41–43^, weak and chiral topological insulators^44–49^, and Dirac and Weyl semimetals^50–55^. By studying $\textrm{SrTiO}_3$ and Si, this work validates ultrafast laser scribing as a versatile approach with the potential to open new avenues for investigating novel quantum phenomena across a wide range of non-natural cleavage planes.

## Methods

$\textbf{SrTiO}_3$** pillars** oriented along the [100] crystallographic axis were fabricated from a commercially grown, lightly electron-doped single-crystal $\textrm{SrTi}_{1{-}x}\textrm{Nb}_x\textrm{O}_3$ (001) substrate (*x = 0.005*) (CrysTec GmbH). The light Nb doping of $\textrm{SrTiO}_3$ substrate provides slight residual bulk conductivity which prevents charging effects during ARPES experiments without affecting the spectral results of surface electronic states. The wafer was cut parallel to the [100] direction using a diamond wire saw (Model 850, Southbay Technology), and subsequently sectioned orthogonally to produce a series of $\textrm{SrTiO}_3$ pillars, approximately 1 mm in height. The pillars were then flipped by $90^\circ$, in order to expose the (100) plane as the top surface of the crystal, and mounted onto a B-doped Si substrate using silver epoxy (EPO-TEK^®^ H20E). A ceramic post was glued onto the top surface of the pillar to assist the cleavage. Grooves were scribed on the lateral surface by scanning back and forth twice along the [001] axis, with a scan velocity of 0.01 mm/s and a pulse energy of 5 ${\mu \textrm{J}}$. The latter corresponds to a peak laser intensity around $10^{13}$ W/cm$^2$. The total fabrication time was *≈ 5* minutes per sample.

**Si pillars** oriented along the [110] crystallographic axis were fabricated from a commercially grown, B-doped single-crystal Si (001) substrate (Siegert Wafer GmbH). The wafer was cut parallel to the [110] direction and subsequently sectioned orthogonally to produce a series of Si pillars, approximately 1 mm in height. The pillars were then flipped by $90^\circ$, in order to expose the (110) plane as the top surface of the crystal, and mounted onto a B-doped Si substrate. A ceramic post was glued onto the top surface of the pillar to facilitate cleavage. Grooves were scribed on the lateral surface by scanning back and forth along the [001] axis, with a scan velocity of 0.02 mm/s and a pulse energy of 3 ${\mu \textrm{J}}$. The total fabrication time was *≈ 3* minutes per sample.

**All grooves** in this study were scribed using a fixed pulse repetition frequency of 250 kHz, at room temperature and ambient pressure. The pulse energy was selected such that the fluence exceeded the ablation threshold, estimated to be of the order of 1 J/cm$^2$^16^. The pulse energy, scan velocity, and number of passes were finely tuned empirically through iterative optimization on several samples. A motorized linear stage (UTS100PPV6, Newport Corporation) controlled by a motion controller (ESP301, Newport Corporation) was used to automatically scan the pillars beneath the laser beam. The entire sample alignment procedure was assisted by two cameras, one positioned to the front of the sample and the other to its side. During laser scribing, a nitrogen flux was applied to the pillars to quickly remove the ablated material from the target and avoid redeposition. Following groove formation, a bending force was applied to cleave the crystal along the scribed line at room temperature. The fabrication process was repeated on more than twenty samples, achieving surfaces of comparable quality to the one presented here, with a success rate of approximately 25%. The success rate was similar for Si and SrTiO$_3$, with the remaining samples displaying smaller homogeneous regions, larger density of redeposited fragments, or non-uniform topography.

**SEM images** were acquired with a Helios 6 HD FIB-SEM (Thermo Fisher Scientific Inc.) system with a nominal pressure below $1 \times 10^{-6}$ mbar and a working distance of approximately 4 mm. An Everhart-Thornley detector was used to collect secondary electrons with an acceleration voltage of 10 kV and a beam current of 0.10 nA for $\textrm{SrTiO}_3$ and 0.40 nA for Si. All SEM images were presented using a grayscale color map, where black and white correspond to the minimum and maximum secondary electron counts, respectively. SEM images were recorded at the Albanova Nanolab, Stockholm.

**AFM images** were acquired using a Dimension Icon AFM system (Bruker Corporation) operated in tapping mode with PeakForce Tapping technology at room temperature in air. A cantilever with a resonant frequency of 150 kHz was employed for imaging all surfaces. The AFM measurements were recorded at the Albanova Nanolab, Stockholm.

**ARPES measurements** were carried out at the MAX IV synchrotron facility in Lund, Sweden, at the Bloch beamline. The photoemission spectra were acquired using a photon energy of 49 eV, linear vertical polarization, i.e., *s*-polarized light, and a beam spot approximately 22 ${\mu \textrm{m}}$ in lateral dimension. The momentum and total energy resolutions were 0.003 Å$^{-1}$ and 10 meV, respectively. The experiment was conducted on a single sample, where two different positions separated by approximately 30 ${\mu \textrm{m}}$ were investigated under the same experimental conditions. The sample was held at a temperature of 18 K during measurements and cleaved *in-situ* with a base pressure below $2 \times 10^{-10}$ mbar. The spectral results for each termination remained stable throughout the measurement period, which lasted approximately one hour per position. All ARPES data were presented using a grayscale color map, where white and black correspond to the minimum and maximum photoelectron counts, respectively.

## Data Availability

The data that support the findings of this study are available from the corresponding author upon reasonable request.
