# Correction: Fabrication of atomically flat cleavage planes with ultrafast laser scribing

**DOI:** 10.1038/s41598-026-64211-6

**Published:** 2026-07-29

**Authors:** Francesco Scali, Wanyu Chen, Magnus H. Berntsen, Cong Li, Jacek Osiecki, Balasubramanian Thiagarajan, Dibya Phuyal, Maciej Dendzik, Oscar Tjernberg

**Affiliations:** 1 Dipartimento di Fisica, Politecnico di Milano, Piazza Leonardo da Vinci 32, 20133 Milano, Italy; 2 Department of Applied Physics, KTH Royal Institute of Technology, 11419 Stockholm, Sweden; 3 MAX IV Laboratory, Lund University, 22100 Lund, Sweden

Correction to: *Scientific Reports* 10.1038/s41598-026-51163-0, published online 14 July 2026

In the original version of the Article, Figure 7 was a duplicated version of Figure 5.

The correct Figure 7 is now updated in the original article.

The original Article has been corrected.

